# Representation of dynamical stimuli in threshold neuron models

**DOI:** 10.1186/1471-2202-12-S1-P376

**Published:** 2011-07-18

**Authors:** Tatjana Tchumatchenko, Theo Geisel, Fred Wolf

**Affiliations:** 1 Max Planck Institute for Dynamics and Self-Organization, 37073 Göttingen, Germany; 2 Bernstein Center for Computational Neuroscience Göttingen, 37073 Göttingen, Germany; 3 The interdisciplinary Collaborative Research Center 889 for Cellular Mechanisms of Sensory Processing, 37075 Göttingen, Germany

## 

**Figure 1:**
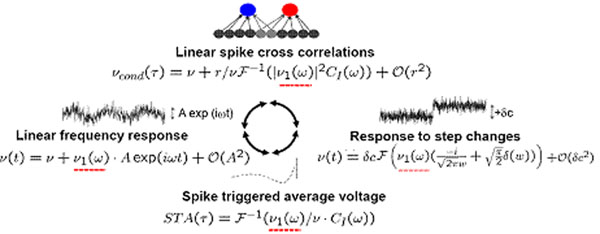
Illustrating the role of the linear frequency response function ν_1_(ω) for the population firing response ν(t) to periodic or step changes of the mean current, the spike triggered average and the pairwise spike correlations ν_cond_(τ) in a pair with a weak input correlation strength r. The dashed red line indicates the presence of ν_1_(ω), C_I_(τ) is the input current correlation function and F denotes the Fourier transform and ν is the stationary firing rate.

A vital function of the mammalian cortex is the processing of dynamical stimuli. These stimuli are encoded in cortical neurons as modifications of the input current, which can be brief, prolonged or periodic, all depending on the type of the sensory stimulus, e.g. [1,2]. While experimental findings can increasingly link sensory stimulation to specific input current modulations, the representation of current stimuli by populations of cortical neurons currently lacks a comprehensive theoretical understanding. In particular, few theories can analytically describe the numerous phenomena related to the processing of dynamical current stimuli, such as pairwise spike correlations and spike triggered average currents (Fig. 1). Even in the simplest integrate and fire model, the complexity of the coupled differential equations allows for tractable analytical results only in specific limiting cases [3,4]. Here, we show how a modified threshold model framework can accurately describe many important features of cortical neurons and provide set of tractable analytical expressions for all quantities of interest shown in Fig.1, such as spike triggered average current, pairwise spike correlations [4,5] and response to dynamical input changes [3,4]. Using this novel model framework, we study how populations of cortical neurons represent dynamical stimuli encoded in the input current and place many important, yet disparate, observations into a common conceptual scheme.
